# Multidrug-Resistant Bacteria in South Atlantic Cetaceans over a Decade of Surveillance

**DOI:** 10.3390/microorganisms14091915

**Published:** 2026-08-30

**Authors:** Felipe da Silva Valente, Karla Renata Kaminski Andrioli, Marcus Adonai Castro da Silva, André Silva Barreto

**Affiliations:** 1 Metabolomics and Applied Biochemistry Laboratory, Federal University of Santa Catarina (UFSC), Florianópolis 88034-000, Brazil; 2 Biodiversity Informatics and Geomatic Laboratory, University of Vale do Itajaí (UNIVALI), Itajaí 88302-901, Brazil; karla.andreoli@univali.br (K.R.K.A.); abarreto@univali.br (A.S.B.); 3 Applied Microbiology Laboratory, University of Vale do Itajaí (UNIVALI), Itajaí 88302-901, Brazil; marcus.silva@univali.br

**Keywords:** antimicrobial resistance, cetaceans, One Health, biological pollution, trophic ecology, sentinel species, ecotoxicology

## Abstract

This decade-long surveillance study (2016–2025) investigates the acquisition of multidrug-resistant (MDR) bacteria in South Atlantic cetaceans to evaluate how ecological niches modulate exposure to biological pollution. Analyzing clinical isolates from stranded cetacean carcasses (*n* = 346), we used Generalized Linear Mixed-Effects Models (GLMMs) to mitigate multi-center analytical biases and compare resistance profiles across coastal and oceanic species. We observed a significant, progressive upward trend in the overall MDR probability over the decade. Although raw MDR was higher in the demersal-feeding *Pontoporia blainvillei* (58.2%) than in the sympatric, water-column-foraging *Sotalia guianensis* (22.0%), multivariate modeling revealed that this difference was primarily driven by the geographic stranding location rather than by intrinsic foraging ecology. High gastrointestinal MDR (73.9%) suggests dietary intake as a primary biological gateway. Demographic modeling revealed a significant sex-based association in *P. blainvillei*, with females facing a higher risk (*p* = 0.009), although the specific ecological or physiological mechanisms underlying this difference remain unknown. High MDR rates in deep-diving *Lagenodelphis hosei* (70.8%) and *Kogia breviceps* (67.9%) suggest that resistant pathogens may reach bathypelagic food webs, potentially via vertical trophic pathways. These findings suggest that spatial environmental contamination, alongside foraging and reproductive ecologies, is a key driver of exposure to the anthropogenic resistome. Because carcass-based sampling inherently targets a diseased or senescent fraction, these high prevalences may overestimate the resistome burden of healthy free-ranging populations. The detection of human pathogens across coastal and offshore habitats indicates persistent deficiencies in terrestrial effluent management, reinforcing cetaceans as One Health sentinels.

## 1. Introduction

Antimicrobial resistance (AMR) is recognized by the World Health Organization (WHO) as one of the top ten threats to global public health, projected to cause 1.91 million attributable deaths worldwide in 2050, accumulating up to 39 million deaths between 2025 and 2050 if current trends persist [1]. The massive and indiscriminate use of antibiotics in human and veterinary medicine has resulted in extensive environmental contamination, transforming natural ecosystems into vast reservoirs of resistance genes [2]. Mitigating this crisis requires a One Health approach that monitors the emergence and spread of these pathogens across environmental matrices, particularly in free-ranging cetacean populations [3].

Coastal and estuarine environments act as ultimate sinks for urban, agricultural, and hospital effluents, becoming active reservoirs for global genetic dissemination rather than merely diluting agents [4]. Within these environments, high bacterial densities, coupled with subclinical concentrations of antibiotics and disinfectants, promote intense selective pressure and facilitate the horizontal transfer of mobile genetic elements [5]. As these populations have no history of direct exposure to clinical therapies, wild animals may acquire multidrug-resistant bacteria through multiple pathways, including contact with fecal-contaminated waters and domestic effluents, though the relative contribution of each route remains unclear for cetaceans [6,7].

Occupying the apex of the food web and possessing long lifespans, marine mammals serve as effective bioindicator sentinels of this kind of ocean pollution, particularly species with high site fidelity that clearly reflect localized contamination from large population centers [8,9]. The exposure of these animals to human-origin pathogens is directly modulated by their ecological niches and foraging strategies, as resistance profiles differ significantly based on diet composition [10]. Cetacean communities in coastal ecosystems exhibit varying degrees of niche overlap, utilizing distinct trophic resources ranging from demersal prey to schooling fish in the water column [11,12,13]. Because environmental resistomes are spatially structured, species with high dependence on demersal foraging are likely to experience different AMR patterns than sympatric species that utilize the water column, even within the same urban ecosystem [10,12].

The present study aims to evaluate the prevalence and temporal dynamics of multidrug-resistant (MDR) bacteria in cetacean carcasses stranded on the Brazilian coast over a decade (2016 to 2025). This study also compares resistance profiles among species with different ecological strategies, using the franciscana (*Pontoporia blainvillei*) and the Guiana dolphin (*Sotalia guianensis*) as biological models to test the hypothesis that localized environmental contamination and coastal sediments serve as the primary reservoirs of bacterial contamination.

## 2. Materials and Methods

### 2.1. Study Area and Sample Collection

**Figure 1:**
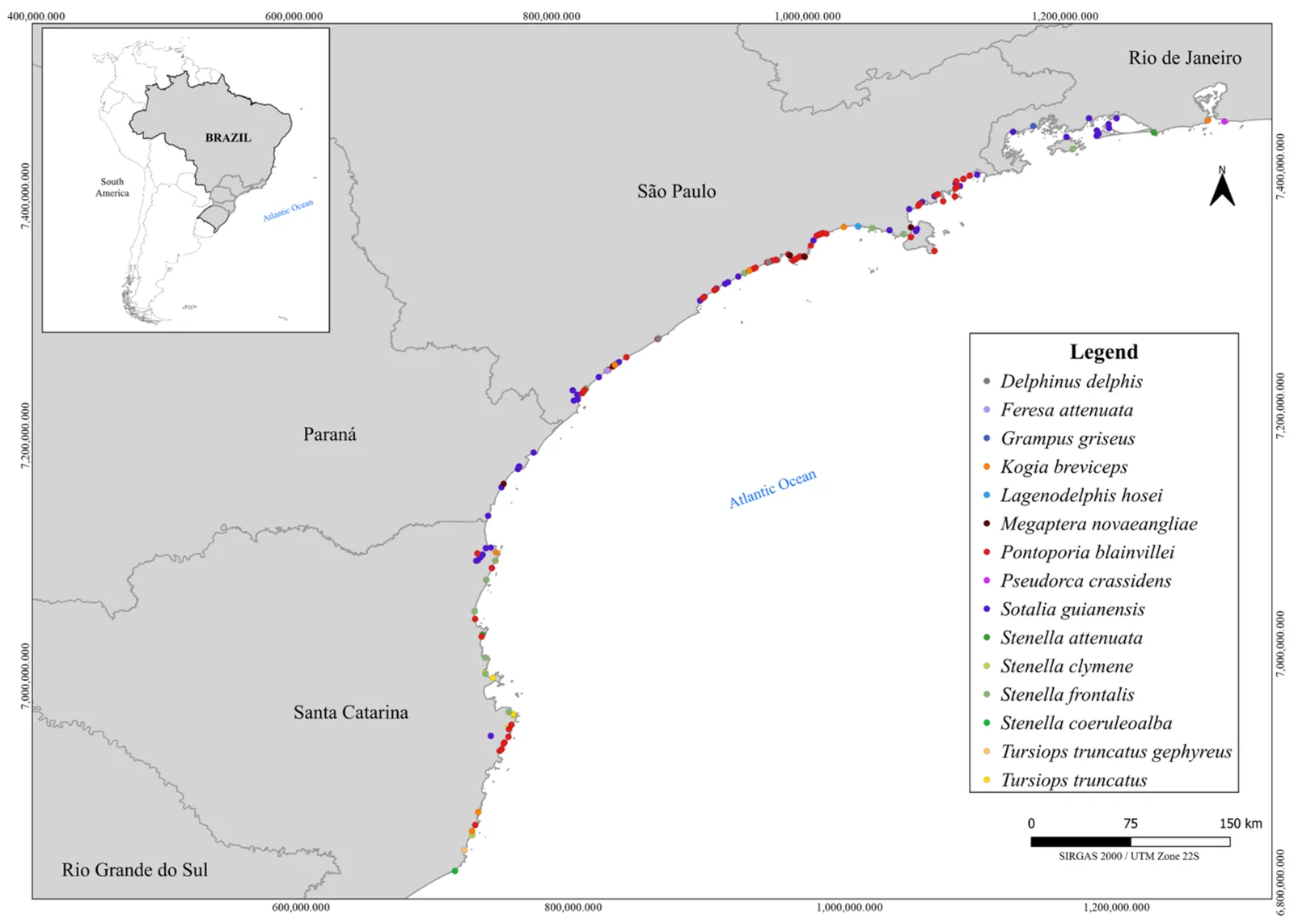
The geographical scope of the study area in the Southwestern Atlantic. The main map illustrates the spatial distribution of cetacean carcasses recovered by the Santos Basin Beach Monitoring Project (PMP-BS) across the Brazilian states of Rio de Janeiro, São Paulo, Paraná, and Santa Catarina. Colored points indicate the exact stranding locations, categorized by species, and sampled for microbiological analysis between September 2016 and November 2025. The inset provides the broader national context for the surveyed region within Brazil. Overlapping points reflect areas with a high density of strandings.

Cetacean carcasses (*n* = 346) used in this study were collected from September 2016 to November 2025 by the Santos Basin Beach Monitoring Project (PMP-BS). The monitoring program is undertaken across four Brazilian states from the South (Santa Catarina, SC; and Paraná, PR) and Southeast (São Paulo, SP; and Rio de Janeiro, RJ) regions. The study area covers a coastal extension of approximately 2013 km, ranging from Laguna in SC (28°29′43.1″ S 48°45′38.5″ W) to Saquarema in RJ (22°56′08.1″ S 42°29′43.9″ W) (Figure 1). Monitoring is conducted through daily and weekly active searches to locate stranded marine megafauna. The PMP-BS is part of the federal environmental licensing conducted by the Brazilian Institute of Environment and Renewable Natural Resources (IBAMA) for oil and gas activities in the Santos Basin. All specimens were collected under the Authorization for Capture, Collection, and Transport of Biological Material issued by IBAMA/Ministry of the Environment (ABIO nos. 640/2015, 1169/2019 and 755/2016).

Necropsies were performed at veterinary facilities separate from the institutions responsible for beach monitoring in each area, in accordance with standard protocols for necropsy of dead marine mammals. To minimize potential post-mortem and environmental contamination, microbiological sampling was strictly limited to fresh or moderately decomposed carcasses (codes 2 and 3) [14]. Code 2 corresponded to fresh carcasses with minimal post-mortem changes, characterized by preserved skin elasticity, intact and moist eyes and mucous membranes, absence of bloating or marked odor, and well-preserved internal organs. Code 3 included carcasses at an early to moderate stage of decomposition, characterized by bloating, moderate odor, epidermal changes, and some post-mortem discoloration, but with internal organs still intact and retaining their normal consistency. During these necropsies, biological samples were aseptically collected by the veterinary team as part of the diagnostic investigation, particularly when macroscopic findings suggested infectious or inflammatory processes. Sampling preferentially targeted intact internal organs and tissues considered diagnostically relevant, without evidence of external exposure, scavenging, or advanced autolysis. Samples included swabs from lesions, physiological fluids (e.g., blood and cavity fluids), and tissue fragments from various organs.

### 2.2. Microbiological Isolation and Antimicrobial Susceptibility Testing (AST)

According to the standardized operating protocols of the PMP-BS, specimens for aerobic bacterial cultures were obtained during necropsies to minimize contamination by the environmental microbiota. Sample collection and transport procedures, as documented in the project’s institutional guidelines, primarily involved the use of sterile swabs immediately preserved in specialized transport media (e.g., Stuart, Cary–Blair, or Amies) or, in cases of suspected systemic infection, direct inoculation into aerobic blood culture bottles. Samples were maintained under refrigeration (2 °C to 8 °C) and transferred to the processing facilities within 24 h.

Given the long-term, large-scale, and multi-center nature of the monitoring program, clinical samples were processed by accredited regional veterinary diagnostic laboratories across the study area. All data on bacterial isolation, taxonomic identification, and Antimicrobial Susceptibility Testing (AST) were retrieved retrospectively from the corresponding laboratory diagnostic reports stored in the institutional database. Based on these historical records, bacterial identification was performed using standard culture media and biochemical profiling, supplemented at specific facilities by automated diagnostic systems (e.g., BD Phoenix™ and Beckman Coulter MicroScan). AST was performed utilizing either the standard Kirby–Bauer disk diffusion method or automated Minimum Inhibitory Concentration (MIC) systems. The interpretation of susceptibility profile, categorically recorded as Susceptible (S), Intermediate (I), or Resistant (R), was performed by the executing laboratories in accordance with the contemporary clinical breakpoints and guidelines established by the Clinical and Laboratory Standards Institute (CLSI) at the time of each diagnostic procedure.

Because raw MIC values and exact disk diffusion zone diameters were not systematically archived in all historical diagnostic reports, it was not feasible to retrospectively re-interpret all isolates using contemporary 2026 CLSI breakpoints. Consequently, the categorical S/I/R interpretations defined by the executing laboratories at the time of diagnosis were retained. These interpretations strictly followed the contemporary guidelines established by the Clinical and Laboratory Standards Institute (CLSI), evolving from historical parameters [15] to the most recent clinical [16] and veterinary [17] protocols. To provide full methodological transparency regarding the evolution of these interpretive criteria over the 10-year surveillance period, a comprehensive summary of the specific CLSI disk diffusion breakpoints (zone diameters in mm) utilized at the beginning (2016) and end (2025) of the study is detailed in Table A3.

### 2.3. Data Curation and Taxonomic Standardization

Raw diagnostic reports were exported from the SIMBA database https://simba.petrobras.com.br (accessed on 15 April 2026). To ensure high data quality and reproducibility, an extensive data-wrangling and standardization process was conducted. Bacterial taxonomy was standardized, and co-isolates from the same sample were appropriately separated. Antimicrobial agent names were translated and standardized to the American Society for Microbiology (ASM) nomenclature using regular expressions (Regex) to eliminate inconsistencies from the raw text.

For high-resolution phenotypic profiling (e.g., MAR Index and correlation matrices), individual antimicrobial agents were maintained. However, for comparative epidemiological risk assessments, the individual antimicrobial agents were systematically grouped into broad pharmacological classes (e.g., Aminoglycosides, 3rd/4th Generation Cephalosporins, Fluoroquinolones), following the Clinical and Laboratory Standards Institute (CLSI) and World Health Organization (WHO) categorization frameworks. Furthermore, anatomical sampling sites were grouped into four major clinical categories by physiological system: Respiratory Tract, Systemic/Internal Organs, Gastrointestinal, and Skin/Superficial.

### 2.4. Antimicrobial Resistance Metrics

Two main metrics were calculated to assess the burden of resistance. First, the Multiple Antibiotic Resistance (MAR) index was calculated for each isolate as the ratio of the number of antibiotics to which the isolate was resistant (a) to the total number of antibiotics against which the isolate was tested (b), formulated as MAR = $\frac{a}{b}$ [18].

Second, Multidrug Resistance (MDR) was strictly defined as acquired non-susceptibility to at least one agent across three or more antimicrobial categories, following international standardized criteria [19]. To avoid sampling bias and ensure robust categorizations, isolates tested against fewer than three antimicrobial categories were excluded from both MAR and MDR calculations.

### 2.5. Statistical Analysis

All data processing, visualization, and statistical analyses were performed using R (version 2026.01.2+418) with the *tidyverse* ecosystem and additional packages for statistical modeling. Descriptive statistics were used to summarize pathogen prevalence, antimicrobial resistance rates, and multidrug resistance (MDR). To reduce the influence of highly unbalanced sample sizes during comparative narratives, descriptive analyses were highlighted for species represented by at least 10 isolates (*n* ≥ 10). To minimize analytical artifacts, resistance profiles were summarized at the examination–pathogen level before statistical analyses.

The Kruskal–Wallis nonparametric test was used to compare Multiple Antibiotic Resistance (MAR) indices across cetacean species that met the minimum sample size criterion (*n* ≥ 10). When the overall test was significant, Dunn’s post hoc multiple comparisons test was performed with a Benjamini–Hochberg adjustment using the *FSA* package. Differences in MDR prevalence between deep/systemic and superficial/tegumentary anatomical categories were assessed using a 2 × 2 contingency table. Pearson’s Chi-square (*χ*^2^) test with Yates’ continuity correction was applied when expected cell frequencies were adequate; otherwise, Fisher’s exact test was used. To investigate potential variations in resistance acquisition, Fisher’s exact test was also used to compare MDR prevalence for specific shared bacterial taxa between hosts (e.g., *Pseudomonas putida* complex and *Escherichia coli*).

Temporal variation in MDR occurrence from 2016 to 2025 was evaluated using a binomial generalized linear model (GLM). For this analysis, MDR-positive and MDR-negative observations were aggregated by year and modeled as binomial counts, with year included as the predictor. Phenotypic co-resistance patterns among the most frequently tested individual antibiotics were evaluated by generating a Spearman rank correlation matrix with the *corrplot* package. To account for multiple testing within the correlation analysis, raw *p*-values were adjusted using the Benjamini–Hochberg False Discovery Rate (FDR) procedure.

Differences in overall phenotypic resistome composition among host species were evaluated using Permutational Multivariate Analysis of Variance (PERMANOVA) with the *vegan* package, based on a Jaccard distance matrix with 999 permutations (excluding fully susceptible isolates to satisfy the metric’s mathematical assumptions). To quantify the ecological risk between the two most representative coastal species (*P. blainvillei* and *S. guianensis*) and to evaluate the influence of host demographics on MDR occurrence, generalized linear mixed-effects models (GLMMs) with a binomial distribution (logit link) were applied using the *lme4* package. In the interspecific model, MDR status was included as the binary response variable; ‘Species’, ‘Age’, and ‘Sex’ were included as fixed effects; and ‘Laboratory’ was included as a random intercept to account for inherent processing variation. A second GLMM was fitted exclusively for *P. blainvillei* to evaluate the associations of ‘Age’ and ‘Sex’ with MDR occurrence, retaining ‘Laboratory’ as a random effect. For resistance-class comparisons between *P. blainvillei* and *S. guianensis*, resistance was summarized at the antimicrobial-class level, and Fisher’s exact test was used to compare the proportions of resistant observations between species. Statistical significance was set at *α* = 0.05 for all tests.

## 3. Results

### 3.1. Prevalence of Multidrug Resistance (MDR) and Global Burden

Bacterial isolates from cetaceans collected over a decade (2016–2025) were evaluated. To ensure a robust categorization, isolates tested against fewer than three antimicrobial categories were excluded from the analysis (*n* = 138). After this filtration and the removal of duplicates, a final dataset of 551 clinical isolates was retained. Applying the strict clinical criteria for Multidrug Resistance (acquired non-susceptibility to ≥3 antimicrobial classes), the analysis demonstrated that the highest MDR prevalences within the main representative cohort (*n* ≥ 15) were concentrated in the pelagic species *Lagenodelphis hosei* (70.8%; 95% CI: 50.8–86.6%) and *Kogia breviceps* (67.9%; 95% CI: 47.6–83.4%), followed by the coastal *P. blainvillei* (58.2%; 95% CI: 51.8–64.3%) and *Tursiops truncatus* (47.4%; 95% CI: 25.2–70.5%) (Table 1).

Beyond this primary cohort, the surveillance also detected critical resistance profiles in species with smaller sample sizes (*n* < 15), which are detailed comprehensively in Table A1. Incidental findings revealed a 100.0% MDR prevalence in *Pseudorca crassidens* (*n* = 10) and 37.5% in the migratory mysticete *Megaptera novaeangliae* (*n* = 8). Given the wide confidence intervals due to these very small sample sizes, these data should be treated strictly as incidental findings rather than as robust population-level estimates. Nonetheless, they underscore the ubiquity of resistant pathogens across distinct cetacean families and foraging guilds in the South Atlantic.

In a crude initial comparison of the two primary coastal species, the raw proportion of MDR isolates in *P. blainvillei* appeared higher than in *S. guianensis*. To determine whether this difference was driven by intrinsic species ecology or by confounding spatial factors, this raw prevalence was further analyzed using multivariate modeling (see Section 3.3).

**Figure 2:**
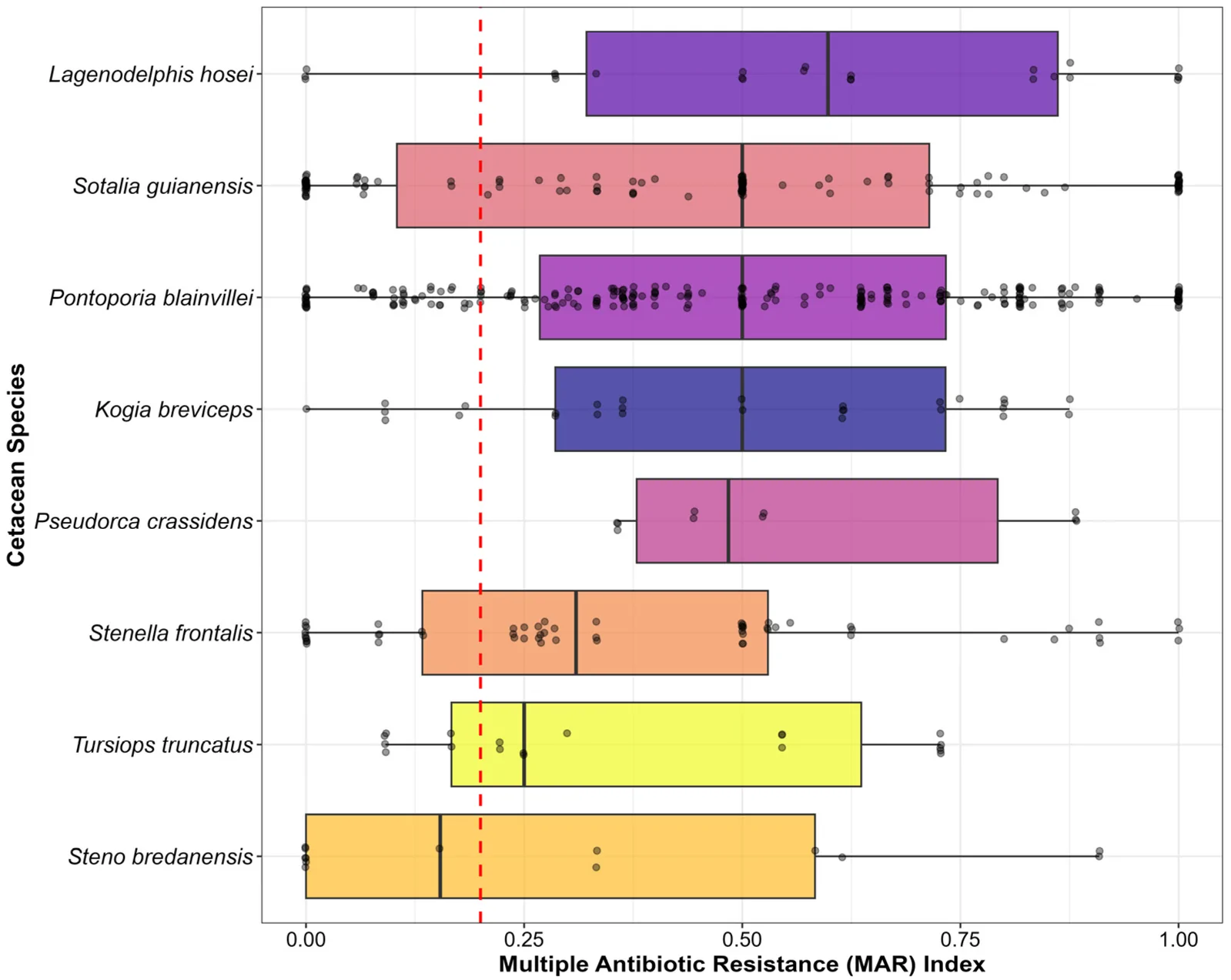
Multiple Antibiotic Resistance (MAR) Index across cetacean species. To ensure robust visual comparisons, only species with representative sample sizes (*n* ≥ 10 isolates) are displayed. The red dashed line indicates the high-risk threshold (MAR > 0.2). Black dots represent outlier values.

The assessment of the overall resistance burden using the MAR Index confirmed a marked heterogeneity among species (Kruskal–Wallis, *χ*^2^ = 16.54; *df* = 7; *p* < 0.020) (Figure 2). However, Dunn’s post hoc analysis (with a Benjamini–Hochberg adjustment) did not yield statistically significant pairwise comparisons (*p* > 0.05), likely due to the adjustment’s high conservatism and interindividual variability. Notably, most cetacean species exhibited median MAR values exceeding the commonly accepted high-risk threshold of 0.2. In addition, the phenotypic resistome composition differed significantly among species (PERMANOVA; *F* = 4.71; *R*^2^ = 0.056; *p* = 0.001), reinforcing the presence of species-specific resistance profiles despite the overall high burden of resistance.

To determine whether the high resistance burden was driven by the host’s specific ecological niche or by the broader coastal circulation of resistant lineages, we compared MDR prevalence within shared pathogenic species between the two coastal hosts. Notably, the *P. putida* complex revealed a significantly higher MDR prevalence in isolates from *P. blainvillei* (29.0%; *n* = 9/31) compared to *S. guianensis* (4.2%; *n* = 1/24) (Fisher’s Exact Test, *p* = 0.031). Furthermore, *E. coli* isolates showed a similar trend, with substantially higher resistance in *P. blainvillei* (71.1%; *n* = 32/45) than in *S. guianensis* (42.9%; *n* = 9/21; *p* = 0.033).

### 3.2. Resistance Analysis by Pharmacological Class

The phenotypic profile stratified by antimicrobial class revealed that pathogens from *P. blainvillei* exhibit statistically higher rates of resistance to classes of critical importance, notably 3rd-/4th-generation cephalosporins (57.4% vs. 32.6%; *p* = 0.003) (Table 2). Resistance to carbapenems was detected exclusively in *P. blainvillei* (24.1%), although the comparative analysis between the two species did not achieve formal statistical significance (*p* = 0.076), primarily due to the limited number of *S. guianensis* isolates tested against this class of last-resort antibiotics.

**Figure 3:**
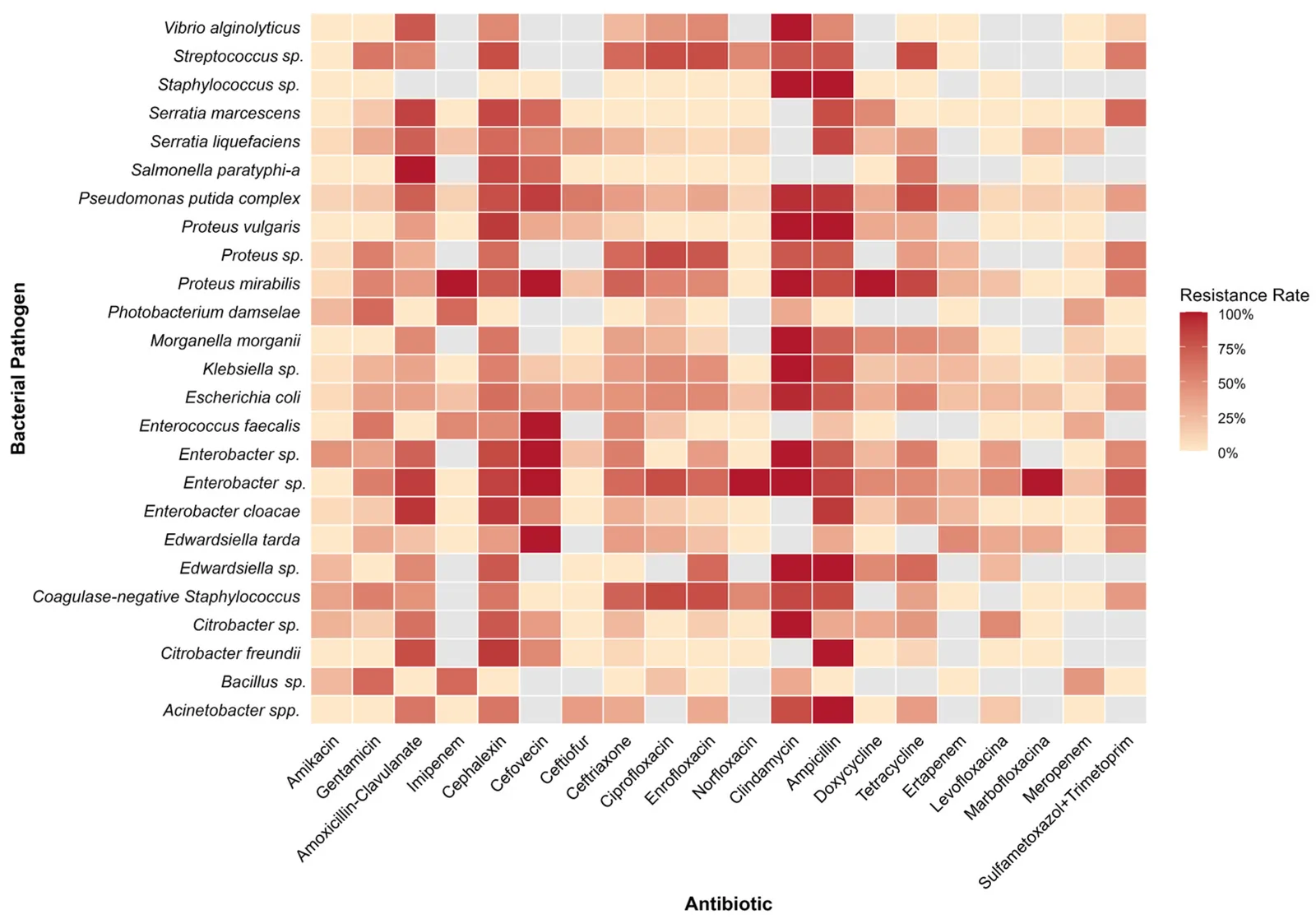
Heatmap of antimicrobial resistance rates across the 25 most frequently isolated bacterial pathogens derived from the entire cetacean cohort (all sampled species). The color gradient illustrates the prevalence of phenotypic resistance, ranging from light peach (0%, fully susceptible) to dark red (100%, fully resistant). Light gray tiles represent non-evaluated combinations (NA) resulting from variable testing panels and standard cascading antimicrobial susceptibility testing (AST) protocols across the longitudinal dataset.

The pathogens predominantly associated with these resistance profiles included *E. coli*, *Pseudomonas aeruginosa*, and *Klebsiella* sp. The heatmap (Figure 3) illustrates the phenotypic resistance profiles and broad tolerance of these specific strains across the evaluated antimicrobial classes.

### 3.3. Temporal Dynamics, Anatomical Distribution, and Demographic Patterns of Multidrug Resistance

**Figure 4:**
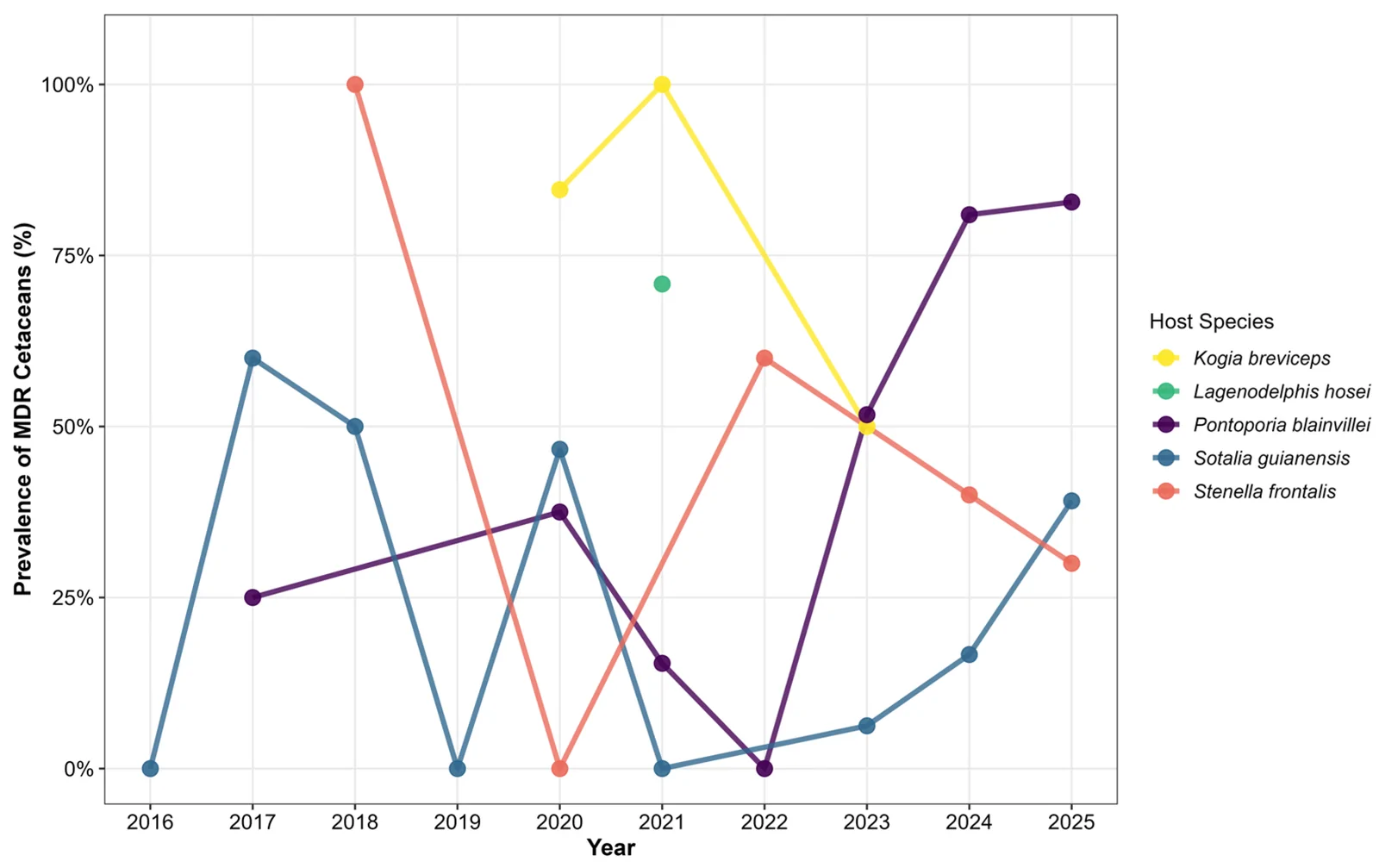
Temporal trend of Multidrug Resistance (MDR) prevalence in the most frequently sampled cetacean species between 2016 and 2025.

MDR prevalence increased significantly and progressively over the study period (Estimate = +0.159; *p* < 0.001), indicating a rising frequency of multidrug resistance in the sampled population (Figure 4). Regarding the origin of the isolates, MDR prevalence was identified in all evaluated compartments: gastrointestinal tract (73.9%), skin/superficial lesions (45.0%), respiratory tract (43.4%), and systemic/internal organs (39.3%). Anatomical stratification showed no statistically significant difference in the probability of isolating MDR pathogens between deep/systemic and superficial infections (*χ*^2^ < 0.001; *df* = 1; *p* = 1.000).

To assess ecological risk while rigorously controlling for confounding variables, a GLMM was utilized. When adjusting for demographic factors (age and sex) and spatial/institutional baseline variability (incorporating ‘Laboratory’ as a random effect, which serves as a direct proxy for the geographic stranding zone, given that specific diagnostic facilities process carcasses strictly from distinct regional stretches of the coastline), the previously observed crude difference in MDR prevalence between *P. blainvillei* and *S. guianensis* lost statistical significance (Adjusted *OR* = 1.008; *p* = 0.99). The substantial variance attributed to the random effect (Variance = 42.18) indicates that the geographic location of the stranding and the processing facility are the primary drivers of MDR risk, overriding species-specific differences.

In the species-specific GLMM for the *P. blainvillei* cohort, demographic analysis revealed a significant sex-driven association. After controlling for regional laboratory variance, male *P. blainvillei* exhibited significantly lower odds of carrying MDR pathogens than females (Estimate = −1.47; *p* = 0.009). Age was not a significant independent predictor of MDR carriage for this species (*p* = 0.818).

### 3.4. Co-Resistance Network

**Figure 5:**
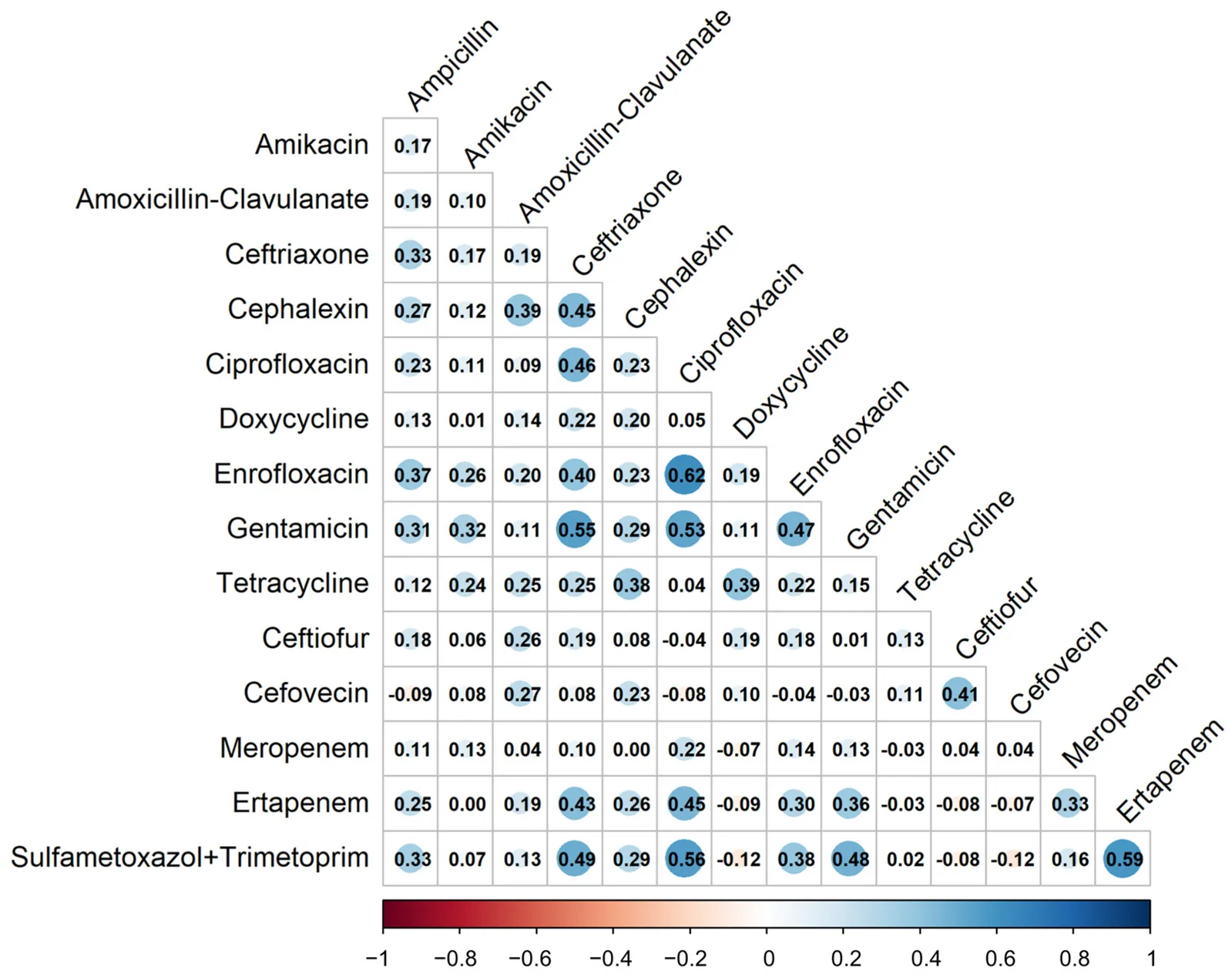
Spearman’s rank correlation matrix illustrating phenotypic co-resistance patterns among the most frequently tested antibiotics. Darker blue circles indicate stronger positive correlations.

The phenotypic co-resistance network analysis, based on Spearman’s rank correlation coefficients (Figure 5), identified significant positive associations both within and between antimicrobial classes. Moderate intra-class cross-resistance was observed among different generations of cephalosporins, such as between cephalexin and ceftriaxone (*r* = 0.46). Notably, the analysis also revealed relevant co-resistance patterns across structurally distinct antimicrobial classes, including positive correlations between sulfamethoxazole-trimethoprim and both ciprofloxacin (*r* = 0.56) and ertapenem (*r* = 0.59).

## 4. Discussion

The present study reveals high rates of multidrug resistance in the South Atlantic marine ecosystem, highlighting the vulnerability of endemic species, such as *P. blainvillei*. The MDR prevalence of 58.2% observed in this coastal species supports the role of small cetaceans as sentinels of ecosystem health in other anthropogenically impacted marine environments. Studies conducted in the Salish Sea and the North and Baltic Seas have shown that harbor porpoises (*Phocoena phocoena*) exhibit substantially higher MDR rates (ranging from 39% to 80%) than sympatric pinniped species, suggesting either increased susceptibility or greater exposure to anthropogenic sources of antimicrobial resistance [12,20].

Despite the relatively small number of isolates available for pelagic species, the high MDR prevalence observed in *L. hosei* (70.8%) and *K. breviceps* (67.9%) is noteworthy and consistent with findings from the Philippines, where 79.17% of bacterial isolates recovered from cetaceans, including *L. hosei* and *K. breviceps*, were classified as multidrug-resistant [21]. The occurrence of MDR in offshore and deep-water species suggests that antimicrobial resistance is not confined to coastal environments and may reflect the dispersal of resistant bacteria and resistance determinants across broader marine ecosystems. The high prevalence of resistance across species occupying distinct ecological niches further supports the role of cetaceans as sentinels of biological pollution, reflecting the dissemination of antimicrobial resistance from human and veterinary sources into the marine environment.

To contextualize the severity of this scenario within a global framework, Table 3 compares the MDR prevalences observed in the present study with those reported in cetacean populations from other ocean basins. Despite inherent methodological variations across epidemiological surveys, a clear global pattern emerges: multidrug resistance in marine mammals is correlated with intense anthropogenic pressure. Regardless of the geographic region, high MDR burdens are consistently driven by shared terrestrial sources, primarily domestic sewage, hospital effluents, and agricultural runoff.

Aquatic environments play a fundamental role in the dissemination of antibiotics and antimicrobial-resistant bacteria across natural ecosystems [2,5,22]. Wastewater treatment systems often fail to fully remove antibiotics and resistant bacteria, allowing their spread into coastal waters, where contamination levels naturally increase due to anthropogenic activity. In this context, estuarine and coastal environments serve as ultimate sinks for agricultural runoff and sewage discharge, acting as active reservoirs for antibiotic metabolites, resistant bacteria, and resistance genes [4,10,12].

Marine sediments act as long-term physical repositories for bacterial communities. These estuarine beds offer conditions such as low oxygen and high nutrient content that support bacterial survival, effectively transforming them into chronic bioreactors for genetic exchange via biofilm formation and horizontal gene transfer [22]. The frequent association of *P. blainvillei* with these areas [13] further increases its exposure to resistant strains.

Historically, environmental monitoring has operated under the assumption that rapid dilution in offshore waters adequately mitigates general pollution risks [23]. However, our findings challenge the paradigm of a pristine pelagic zone. Our detection of high MDR rates in deep-ocean cetaceans, notably *L. hosei* (70.8%) and *K. breviceps* (67.9%), raises serious concerns about offshore contamination pathways reaching the outer continental shelf and slope. The high MDR levels observed in pelagic cetaceans may reflect vertical connectivity within the marine ecosystem rather than direct proximity to coastal contamination. Diverse and potentially mobile antimicrobial resistance genes (ARGs) have been detected in mesopelagic and bathypelagic waters, including depths exceeding 1000 m [24]. Marine microplastics can also harbor and enrich resistant bacteria and ARGs, providing a potential mechanism for their dispersal across marine environments [22,25]. The pathways through which oceanic cetaceans acquire resistant bacteria remain unclear. Some authors have proposed that contact with surface waters during respiration could represent a route of exposure for deep-diving marine mammals [21,26,27]. We hypothesize that trophic connectivity may contribute to exposure: deep-diving cetaceans feed on mesopelagic fishes and squids that undergo diel vertical migrations [28], potentially facilitating the transfer of resistant bacteria or resistance determinants across depth gradients. Further investigation of mesopelagic prey resistomes is needed to test this hypothesis. Evidence from Brazilian waters further supports the exposure of deep-diving cetaceans to clinically relevant resistant bacteria. The recent identification of hypervirulent, carbapenemase-producing (NDM-1) *E. coli* lineages in *K. breviceps* specimens from Brazil [29] reveals that critically important human pathogens can persist in the marine environment and reach the food web of deep-diving species. This phenomenon clearly indicates that biological pollution extends into mesopelagic regions via ocean currents or the displacement of infected prey.

The taxonomic profile of the isolates reinforces the hypothesis of anthropogenic pollution in the coastal ecosystem. The predominance of high-priority human clinical pathogens, such as *E. coli*, *P. aeruginosa*, and *Klebsiella* sp., provides evidence of the direct impact of inadequately treated sewage discharge on marine habitats [4,10,30]. Wild animals acquire multidrug-resistant bacteria primarily through exposure to urban effluents and agricultural runoff, both of which are major drivers of the environmental resistome [10,31]. This risk scenario is shared by other cetacean species in Brazil that exhibit high resistance to advanced cephalosporins and priority pathogens [3,32]. Chronic exposure of marine mammals to these pathogens heightens the risk of zoonotic transmission to professionals involved in necropsies and rehabilitation, requiring rigorous occupational biosafety protocols to prevent associated infections [33].

This phenomenon demonstrates that the microbiota of wild species reflects the sanitary pressure exerted by adjacent terrestrial human activities. Although current global surveillance systems still primarily focus on clinical and agricultural settings, wildlife can serve as valuable sentinels for the abundance and distribution of terrestrial pathogen pollution, reflecting both ecosystem health and the effectiveness of mitigation strategies [8,9,10,33]. These findings consolidate cetaceans as natural reservoirs and epidemiological sentinels under the One Health paradigm. Curiously, the lack of statistically significant differences in resistance to fluoroquinolones and penicillins between the two main coastal species suggests that contamination by these classes is already widespread in the water column, affecting both demersal foraging species and those restricted to the pelagic environment.

Additionally, the binomial regression analysis from 2016 to 2025 demonstrated that the probability of MDR is not on a stable plateau; rather, it shows a significant, progressive upward trend. This escalating trend suggests that antimicrobial resistance is actively becoming more prevalent among the sampled cetaceans. This dynamic suggests that the coastal biota is experiencing increasing selective pressure, thereby establishing cetaceans as sentinels of a rapidly deteriorating microbial environment. Persistent deficiencies in basic sanitation policies ensure a continuous influx of resistance genes and pharmaceutical residues, thereby increasing selective environmental pressure year after year [10,22,34].

These escalating temporal dynamics in the marine environment mirror the alarming rise in antimicrobial resistance observed worldwide among critical zoonotic pathogens, highlighting a global macro-epidemiological trend [35]. While temporal tracking of antimicrobial resistance has been documented in marine mammals from the Northern Hemisphere [7,36], the novelty of the present study lies in translating a robust, decade-long epidemiological baseline to South Atlantic cetacean sentinels. While Whole-Genome Sequencing (WGS) has been extensively shown to facilitate the identification of AMR determinants and to predict phenotypic resistance [35,37,38], its systematic application in marine mammal conservation remains in its infancy [29,32]. By establishing this decade-long phenotypic baseline, our study contributes valuable baseline data to inform future genomic surveillance in the South Atlantic.

When the analysis was restricted to the two most abundant coastal species, our mixed-model analysis revealed that controlling for spatial and institutional variance (the laboratory center effect) nullified the raw disparity in MDR risk between *P. blainvillei* and *S. guianensis*. This suggests that the environmental contamination level of the specific coastal zone where the animal resides and strands influences the resistome burden more heavily than the species’ inherent foraging strategy. In highly urbanized coastal sinks, pervasive environmental pollution appears to outweigh fine-scale ecological niche partitioning. This pattern corroborates recent observations that aquatic mammals face ubiquitous selective pressures due to chronic exposure to anthropogenic matrices [4,8]. The scientific literature indicates that *P. blainvillei* is an opportunistic predator with trophic plasticity in shallow coastal waters [13,19]. While stable isotope analyses indicate a high degree of isotopic overlap and resource sharing between *P. blainvillei* and the sympatric *S. guianensis* [19,39], their fine-scale habitat use diverges. *S. guianensis* exhibits a broader δ^13^C range, suggesting foraging across varied habitats within the same coastal system [19]. In contrast, *P. blainvillei* exhibits a narrower isotopic niche, suggesting more restricted spatial exploration [19]. Because antimicrobial resistance profiles in wildlife are directly modulated by diet composition and foraging strategies [10,34], the restricted, localized coastal foraging behavior of *P. blainvillei* may continuously expose it to localized points of effluent deposition. Dietary assessments across the species’ southern and southeastern range confirm that while *P. blainvillei* feeds throughout the water column as an opportunistic predator, it frequently consumes demersal prey (e.g., sciaenidae fishes and cephalopods) associated with unconsolidated coastal and estuarine substrates [11,13,19,40]. This substantial intake of bottom-dwelling prey could increase exposure to sediment-bound resistomes.

The ecological vulnerability of this species to anthropogenic pollution is further evidenced by its close association with physical matrices, as *P. blainvillei* exhibits the highest rate of marine debris ingestion (17.9%) along the southern coast of Brazil [41]. The accumulation of chemical pollutants in this debris and surrounding sediments probably exerts further environmental pressures. Recent metabolomic analyses revealed substantial contamination by phthalate esters (PAEs), endocrine-disrupting plastic additives, in the blubber of *P. blainvillei* from the Santa Catarina coast, indicating chronic exposure to plastic polymers in these coastal sinks [42]. While neonates are inherently vulnerable to opportunistic pathogens (e.g., *P. aeruginosa*) due to physiological immune immaturity [30], the chronic bioaccumulation of potentially immunosuppressive chemicals represents an additional factor that may compromise immune defenses in the wider population [8]. This concurrent exposure to chemical pollutants may exert selective pressures that drive bacterial multidrug resistance through genetic co-selection mechanisms [2,22]. This dynamic is similarly hypothesized in freshwater ecosystems, where metallic residues drive the selection of antibiotic-resistant microbiota in Amazon basin cetaceans [3].

Consistent with this ecological framework, our intra-pathogen analysis supports the hypothesis that sediment may be a major reservoir of biological pollution. The *P. putida* complex, a group frequently recovered from organically enriched aquatic environments [43,44], exhibited significantly greater multidrug resistance (29.0%) when isolated from *P. blainvillei* compared to *S. guianensis* (4.2%; *p* = 0.031). Similarly, *E. coli* isolates mirrored this trend (71.1% vs. 42.9%; *p* = 0.033). This intraspecific difference suggests that the worsening of the resistance profile may be linked to foraging strategy and permanence of *P. blainvillei* in a niche contaminated by various anthropogenic matrices. As wild populations are increasingly forced to forage on resources contaminated by human effluents, their native microbial composition is altered by exposure to environmental resistomes. Indeed, antimicrobial resistance profiles in wildlife are closely tied to feeding strategies, and foraging behavior in proximity to human activities significantly increases the acquisition of resistant pathogens through trophic levels [10,31].

Pathogens from *P. blainvillei* exhibited statistically higher rates of resistance to critical human antibiotics, including third- and fourth-generation cephalosporins (57.4% vs. 31.2% in *S. guianensis*; *p* = 0.001). Resistance to these advanced cephalosporins in *E. coli* is a growing global concern, largely driven by enzymes such as extended-spectrum beta-lactamases (ESBLs) that are increasingly reported in wildlife populations [10,45]. This observed increase in resistance to last-generation cephalosporins and aminoglycosides in coastal cetaceans is a documented trend in other regions with high anthropogenic pressure, suggesting shared exposure to terrestrial contamination sources [7]. High and disparate prevalences of multidrug resistance among sympatric marine mammals in urban ecosystems reflect a vast environmental pool of resistant microorganisms, modulated by intrinsic differences in habitat use among species [12]. The implications of this scenario (57.8% MDR in *P. blainvillei*) become evident when compared with marine mammal indices in regions with broader coverage of basic sanitation (e.g., 37.2% MDR reported in the North and Baltic Seas; [20]). This contrast highlights the potential impact of selective pressures in the Brazilian coastal environment, likely aggravated by regional deficiencies in effluent management.

Anatomical analysis demonstrated the widespread occurrence of MDR pathogens among the evaluated cetaceans, with no statistically significant difference in the colonization of superficial wounds (45.0%) compared with the infection of deep systemic tissues (39.3%). However, the high prevalence of MDR in the gastrointestinal tract (73.9%) underscores the potential importance of the trophic route in acquiring antimicrobial-resistant bacteria. This anatomical hotspot suggests that ingestion of contaminated demersal prey, incidental sediment intake, and marine debris may represent relevant pathways for exposure to MDR bacteria and environmental reservoirs of antimicrobial resistance. On the other hand, the prevalence of multidrug-resistant isolates in the respiratory tract (43.4%) suggests that cetaceans may be contaminated when surfacing to breathe. The air–water interface acts as a biological and chemical sink, known as the sea surface microlayer (SML), which is highly enriched in dissolved organic matter, lipids, and microbiota, concentrating microorganisms at levels significantly higher than those in the underlying pelagic seawater [26,27].

Once inhaled during surface respiration or ingested, these opportunistic pathogens can breach the epithelial barrier, potentially leading to generalized sepsis. The lethality of this clinical progression resulting from bacterial respiratory infection on the Brazilian coast has been documented in *P. blainvillei* neonates [30]. This fatal systemic failure scenario has also been documented in calves of the same species when these infections involve multidrug-resistant strains carrying critical genes of the highest human priority [32].

Furthermore, the demographic analysis identified a significant sex-specific disparity in *P. blainvillei*, with males showing significantly lower odds of MDR carriage than females (*p* = 0.009). Although the mechanisms underlying this association cannot be established definitively from our retrospective data, several non-mutually exclusive biological and ecological factors may contribute to this pattern. First, sex-related variations in hormones, genetics, and immune responses can alter host–microbiome interactions and bacterial colonization patterns [46]. Because bacterial taxa differ in their intrinsic and acquired resistance profiles, these physiological differences could drive variations in MDR carriage.

Differential environmental exposure may also play a role in this pattern, since Franciscana dolphins exhibit restricted movements and high site fidelity, and some degree of small-scale spatial segregation between sexes has been reported in southern Brazil [47], a pattern also documented in other coastal odontocetes [48]. Such sex-related differences in habitat use could potentially result in differential exposure to localized environmental reservoirs of antimicrobial resistance. However, there is currently no evidence that female *P. blainvillei* preferentially occupy more anthropogenically impacted habitats. Ultimately, the mechanisms underlying this female-biased pattern remain unclear. While it may reflect a complex interplay among host physiology, immunology, and fine-scale spatial dynamics, there are currently no direct behavioral or physiological data to explain this disparity. Therefore, future targeted studies are necessary to elucidate the drivers of sex-specific MDR carriage in this species.

The phenotypic correlation matrix revealed a strong co-occurrence of resistance profiles, suggesting potential co-resistance mechanisms in the South Atlantic Ocean. While cross-resistance among different generations of cephalosporins (e.g., cephalexin and ceftriaxone) is predictable due to their shared targets and mechanisms of action, the occurrence of inter-class co-resistance, such as between sulfamethoxazole–trimethoprim and ciprofloxacin or ertapenem, is a major concern. These correlations suggest genetic co-selection, in which resistance determinants for distinct antimicrobial classes are physically linked on mobile genetic elements (e.g., plasmids, integrons) that circulate in the marine environment [2,5]. This reinforces the widespread dissemination of multidrug-resistant bacterial profiles in marine ecosystems, indicating that resistance in this environment is not sporadic or limited to individual drugs. Rapid changes in bacterial susceptibility in aquatic ecosystems are driven by the continuous influx of effluents, which act as biologically active reactors for the dissemination of resistance genes [5,22]. Recent global mappings confirm that genes conferring resistance to critical classes, such as beta-lactams, are frequent in coastal waters exposed to urban and port discharges [4]. In our dataset, MAR Index values greater than 0.20 indicate exposure to environments heavily impacted by domestic, industrial, and hospital effluents, as observed in cetaceans from other tropical regions [7,18,21]. The progressive escalation of multidrug resistance in marine mammals is a form of environmental degradation driven by the selective pressure of continuous, diffuse discharges [7,36].

Although susceptibility to carbapenems remains 100% in cetacean populations in regions such as the Philippine archipelago [21], our analysis revealed a statistically significant level of carbapenem resistance (24.1%) in *P. blainvillei*. This finding, alongside recent reports of last-resort resistance genes in the marine megafauna of the South Atlantic [29,32], indicates the emergence of highly critical pathogens in coastal ecosystems. This co-selection phenomenon implies that exposure of coastal microbiota to a single environmental residue can trigger genetic cascades that confer simultaneous resistance to critically important hospital antibiotics [2,22], linking deficiencies in urban sanitation to the morbidity of marine megafauna within the One Health paradigm.

Despite the robustness and temporal scale of the findings, this study presents limitations inherent to marine mammal research. First, compiling longitudinal, multicenter antimicrobial susceptibility data over a decade entails inherent methodological heterogeneity. Shifts in CLSI breakpoints over time and the utilization of different diagnostic methods (e.g., automated MIC systems versus Kirby–Bauer disk diffusion) across participating laboratories may introduce baseline variability. Furthermore, the retrospective nature of commercial AST reports inherently leads to variable testing panels across isolates. For instance, last-resort antibiotics such as carbapenems were tested less frequently in typically susceptible cohorts (e.g., *S. guianensis*) under standard cascading AST protocols, thereby limiting sample sizes for comparisons within specific drug classes.

Additionally, sampling based exclusively on carcasses introduces stranding bias, which may overestimate the prevalence of multidrug resistance. Stranded individuals frequently represent an immunosuppressed, diseased, or senile fraction of the population and do not accurately reflect the microbiota of healthy free-ranging animals [12,36]. Therefore, the prevalences reported herein must be interpreted with caution, as they reflect the resistome burden of a compromised fraction of the population, which inherently limits their direct generalizability to the broader, healthy, free-ranging population. Although collection was restricted to fresh or early-decomposition carcasses (codes 2 and 3), the time elapsed between collection and necropsy may affect the recovery of fastidious bacteria and alter the native microbial composition [12]. Another limitation lies in the absence of paired environmental samples (water and estuarine sediment). While the transfer of pathogens from anthropogenic effluents to *P. blainvillei* is supported by the species’ niche ecology, the lack of environmental sequencing of the habitat restricts direct proof of this transmission pathway [12,22]. To overcome these barriers, future studies must shift from phenotypic microbiology to molecular approaches. Coupling metagenomics and Whole-Genome Sequencing [10,12,22] with stable isotope tracking of host δ^13^C and δ^15^N [19,48] will provide high-resolution insights to definitively establish direct source-pathogen linkages between terrestrial effluent sinks and the microbiomes of coastal marine megafauna. The identification of specific Antimicrobial Resistance Genes (ARGs) and the phylogenetic tracking of clonal lineages [10,12,29] will provide high-resolution insights into pathogen flow and elucidate the genetic links between terrestrial anthropogenic sources and the microbiomes of coastal marine megafauna.

## 5. Conclusions

This decade-long surveillance demonstrates that multidrug-resistant (MDR) bacteria carrying determinants conferring resistance to critical human antibiotics are widespread among South Atlantic cetaceans, reflecting chronic deficiencies in regional wastewater management. Importantly, our mixed-model analysis indicates that environmental exposure in highly impacted coastal zones outweighs fine-scale ecological niche differences in driving MDR acquisition. Demographic analyses revealed that female *P. blainvillei* face higher odds of MDR carriage (*p* = 0.009). The specific ecological or physiological mechanisms driving this difference remain unknown and warrant further targeted investigations. Furthermore, the detection of high rates of resistance in deep-diving species (*L. hosei* and *K. breviceps*) challenges the assumption of a pristine offshore environment, suggesting that vertical trophic pathways may transport pathogens into deep-ocean food webs. Given the inherent bias of carcass-based sampling, which tends to capture compromised individuals and limits direct generalizability to healthy populations, wildlife surveillance must be integrated into national One Health plans alongside genomic and environmental monitoring. Mitigating this biological pollution requires the urgent implementation of advanced wastewater treatment infrastructure in coastal municipalities.

## Figures and Tables

**Table 1:** Prevalence of multidrug resistance (MDR) among marine mammal species sampled in the South Atlantic Ocean. MDR was defined as acquired non-susceptibility to at least one agent in three or more antimicrobial categories.

Species	Total Isolates (*n*)	MDR Prevalence (%)	95% CI
Main Cohort (*n* ≥ 15)			
*Lagenodelphis hosei*	24	70.8	48.8–86.6
*Kogia breviceps*	28	67.9	47.6–83.4
*Pontoporia blainvillei*	251	58.2	51.8–64.3
*Tursiops truncatus*	19	47.4	25.2–70.5
*Stenella frontalis*	58	36.2	24.3–49.9
*Sotalia guianensis*	118	22.0	15.1–30.8

**Table 2:** Comparative phenotypic resistance to major antimicrobial classes between the two most frequently sampled coastal cetaceans (*Pontoporia blainvillei* and *Sotalia guianensis*).

Antimicrobial Class	*P. blainvillei* % (*n*/*N*)	*S. guianensis* % (*n*/*N*)	*p*-Value
1st/2nd Gen Cephalosporins	83.9 (161/192)	75.7 (28/37)	0.241
3rd/4th Gen Cephalosporins	57.4 (116/202)	32.6 (15/46)	0.003
Beta-lactam/Inhibitor	63.9 (122/191)	65.5 (36/55)	0.874
Fluoroquinolones	46.6 (96/206)	31.4 (16/49)	0.081
Carbapenems	24.1 (7/29)	0.0 (0/15)	0.076

**Table 3:** Comparison of multidrug resistance (MDR) prevalence and primary environmental drivers in marine mammals across distinct geographic regions.

Geographic Region	Cetacean Species Assessed	MDR Prevalence (%)	Primary Resistance Drivers Suggested	Reference
Southwestern Atlantic (Brazil)	*Lagenodelphis hosei*, *Kogia breviceps*, *Pontoporia blainvillei*, *Tursiops truncatus*, *Stenella frontalis*, *Sotalia guianensis*	58.2% (*P. blainvillei*); 70.8% (*L. hosei*); 67.9% (*K. breviceps*); 22.0% (*S. guianensis*)	Exposure to regional terrestrial effluents across stranding locations; localized coastal contamination overriding fine-scale foraging niches (*P. blainvillei*/*S. guianensis*); vertical trophic pathways (*L. hosei*/*K. breviceps*).	Present Study
North and Baltic Seas	*Phocoena phocoena*	80.0% (MDR among *E. coli* isolates from this species); 39.4% overall prevalence of resistant *E. coli* in marine mammals	Environmental dissemination of anthropogenic origin	Gross et al. [20]
Philippine Archipelago	8 species sampled (MDR isolates recovered specifically from *Kogia breviceps*, *Globicephala macrorhynchus*, *Lagenodelphis hosei*, *Peponocephala electra*)	79.2% (among Enterobacteriaceae isolates)	Exposure to highly polluted water bodies receiving domestic, industrial, and hospital sewage	Obusan et al. [21]
Salish Sea, Washington (USA)	*Phocoena phocoena*	39.0% (MDR among tested porpoises); 52.0% of porpoises resistant to ≥1 antibiotic; 10% of total isolates with MAR index > 0.20	Land-to-sea transfer of pathogens via agricultural runoff, sewage/wastewater effluents, and aquaculture	Norman et al., [12]
Indian River Lagoon, Florida (USA)	*Tursiops truncatus*	88.2% (overall resistance to ≥1 antibiotic); MAR index > 0.20 for 4 major pathogens	Direct terrestrial discharge via septic tanks and human-made drainage canals	Schaefer et al. [7]

## Data Availability

The datasets generated and analyzed during the current study are publicly available at https://simba.petrobras.com.br (accessed on 15 April 2026).

## Abbreviations

The following abbreviations are used in this manuscript:

ABIO	Authorization for Capture, Collection, and Transport of Biological Material
AMR	Antimicrobial Resistance
ARG	Antimicrobial Resistance Gene
ASM	American Society for Microbiology
AST	Antimicrobial Susceptibility Testing
CI	Confidence Interval
CLSI	Clinical and Laboratory Standards Institute
ESBL	Extended-Spectrum Beta-Lactamase
FDR	False Discovery Rate
GLMM	Generalized Linear Mixed-Effects Model
IBAMA	Brazilian Institute of Environment and Renewable Natural Resources
MAR	Multiple Antibiotic Resistance
MDR	Multidrug Resistance
MIC	Minimum Inhibitory Concentration
NDM-1	New Delhi metallo-beta-lactamase 1
OR	Odds Ratio
PAE	Phthalate Esters
PERMANOVA	Permutational Multivariate Analysis of Variance
PMP-BS	Santos Basin Beach Monitoring Project
SML	Sea Surface Microlayer
WGS	Whole-Genome Sequencing
WHO	World Health Organization

## Appendix A

### Appendix A.1

**Table A1:** Comprehensive prevalence of multidrug resistance (MDR) across all marine mammal species sampled in the South Atlantic Ocean (2016–2025).

Species	Total Isolates (*n*)	MDR ^1^ Prevalence (%)
*Pontoporia blainvillei*	251	58.2
*Sotalia guianensis*	118	22.0
*Stenella frontalis*	58	36.2
*Kogia breviceps*	28	67.9
*Lagenodelphis hosei*	24	70.8
*Tursiops truncatus*	19	47.4
*Steno bredanensis*	13	30.8
*Pseudorca crassidens*	10	100.0
*Megaptera novaeangliae*	8	37.5
*Stenella attenuata*	7	28.6
*Tursiops truncatus gephyreus*	5	0.0
*Delphinus delphis*	3	0.0
*Grampus griseus*	3	0.0
*Stenella clymene*	3	0.0
*Feresa attenuata*	1	100.0
*Stenella coeruleoalba*	1	0.0
Total Global	551	-

^1^ MDR was strictly defined as acquired non-susceptibility to at least one agent in three or more distinct antimicrobial categories [16]. To avoid sampling bias, isolates tested against fewer than three antimicrobial categories were excluded from this calculation. Prevalence percentages for species with small sample sizes (*n* < 15, e.g., *Pseudorca crassidens*, *Megaptera novaeangliae*, etc.) are reported here for comprehensive epidemiological transparency; however, these rates should be interpreted with caution due to the wide confidence intervals inherent in limited sampling.

### Appendix A.2

**Table A2:** Significant phenotypic co-resistance patterns (Spearman’s rank correlation coefficient, *r* ≥ 0.40) among the most frequently tested antibiotics.

Antibiotic Pair	Spearman Coefficient (r)
Ciprofloxacin–Enrofloxacin	0.62
Ertapenem–Trimethoprim–Sulfamethoxazole	0.59
Ciprofloxacin–Trimethoprim–Sulfamethoxazole	0.56
Ceftriaxone–Gentamicin	0.55
Ciprofloxacin–Gentamicin	0.53
Ceftriaxone–Trimethoprim–Sulfamethoxazole	0.49
Gentamicin–Trimethoprim–Sulfamethoxazole	0.48
Enrofloxacin–Gentamicin	0.47
Ceftriaxone–Ciprofloxacin	0.46
Ciprofloxacin–Ertapenem	0.45
Ceftriaxone–Cephalexin	0.45
Ceftriaxone–Ertapenem	0.43
Cefovecin–Ceftiofur	0.41

### Appendix A.3

**Table A3:** Evolution of Clinical and Laboratory Standards Institute (CLSI) disk diffusion interpretative criteria (zone diameters in mm) utilized during the decade-long surveillance (2016–2025).

Bacterial Group	Antimicrobial Agent	CLSI Standard Year	Susceptible (S)	Intermediate (I)	Resistant (R)
Enterobacteriales (Changed Breakpoints)	Amikacin	2016	≥15	13–14	≤12
		2025	≥20	17–19	≤16
	Gentamicin	2016	≥15	13–14	≤12
		2025	≥18	15–17	≤14
	Cephalexin	2016	≥15	13–14	≤12
		2025	≥22	-	<22
	Ceftriaxone	2016	≥23	20–22	≤19
		2025	≥25	22–24	≤21
	Ciprofloxacin	2016	≥21	16–20	≤15
		2025	≥26	22–25	≤21
	Enrofloxacin	2016	≥21	17–20	≤16
		2025	≥22	18–21	≤17
	Levofloxacin	2016	≥17	14–16	≤13
		2025	≥26	17–20	≤21
Enterobacteriales (Unchanged 2016–2025)	Amoxicillin–clavulanate	All years	≥18	14–17	≤13
	Ampicillin	All years	≥17	14–16	≤13
	Imipenem	All years	≥23	20–22	≤19
	Meropenem	All years	≥23	20–22	≤19
	Ertapenem	All years	≥22	19–21	≤18
	Cefovecin	All years	≥23	20–22	≤19
	Ceftiofur	All years	≥21	18–20	≤17
	Norfloxacin	All years	≥17	13–16	≤12
	Tetracycline	All years	≥15	12–14	≤11
	Clindamycin	All years	≥21	15–20	≤14
	Trimethoprim–Sulfamethoxazole	All years	≥16	11–15	≤10
Specific Organism Standards	Amikacin	*P. aeruginosa*	≥17	15–16	≤14
	Gentamicin	*P. aeruginosa*	≥15	13–14	≤12
	Imipenem	*P. aeruginosa*	≥19	16–18	≤15
	Meropenem	*P. aeruginosa*	≥19	16–18	≤15
	Ciprofloxacin	*Salmonella* sp.	≥31	21–30	≤20
	Doxycycline	*S. aureus*	≥19	16–18	≤15
	Ampicillin	*Enterococcus*	≥17	-	≤16
	Tetracycline	*Enterococcus*	≥19	15–18	≤14
	Doxycycline	*Streptococcus*	≥28	25–27	≤24
	Tetracycline	*Streptococcus*	≥23	19–22	≤18
	Clindamycin	*Streptococcus*	≥19	16–18	≤15
	Trimethoprim–Sulfamethoxazole	*Streptococcus*	≥19	16–18	≤15

Note: For antimicrobials with evolving guidelines, the initial (2016) and final (2025) disk diffusion breakpoints are presented to illustrate temporal methodological shifts. Interpretative criteria were meticulously extracted from the historical CLSI M100-S25 guideline (2015) [15], the current clinical CLSI M100 36th ed. (2026) [16], and the CLSI Veterinary 7th ed. (2024) [17].
